# Protocol: Strategies to Enhance Inclusion in Informed Consent Practice for People With Vision and/or Hearing Support Needs: A Systematic Review

**DOI:** 10.1002/cl2.70065

**Published:** 2025-09-29

**Authors:** Fleur O'Hare, Sujani Thrimawithana, Aimee Clague, Eden G. Robertson, David Foran, Caroline Ondracek, Camille Paynter, Tessa Saunders, Lauren N. Ayton

**Affiliations:** ^1^ Department of Optometry and Vision Sciences, Melbourne School of Health Sciences University of Melbourne Melbourne Victoria Australia; ^2^ Centre for Eye Research Australia Melbourne Victoria Australia; ^3^ University Library University of Melbourne Melbourne Victoria Australia; ^4^ Discipline of Paediatrics and Child Health, School of Clinical Medicine UNSW Medicine & Health, UNSW Sydney Sydney New South Wales Australia; ^5^ Stem Cell Medicine Group Children's Medical Research Institute Sydney New South Wales Australia; ^6^ Equalus Advisory Noosa Heads Queensland Australia; ^7^ Library Services Royal Children's Hospital Melbourne Melbourne Victoria Australia; ^8^ Department of Audiology and Speech Pathology, Melbourne School of Health Sciences University of Melbourne Melbourne Victoria Australia; ^9^ Evaluation and Implementation Science Unit, Melbourne School of Population and Global Health University of Melbourne Melbourne Victoria Australia; ^10^ Department of Surgery (Ophthalmology), Melbourne Medical School University of Melbourne Melbourne Victoria Australia

## Abstract

An equitable and accessible informed consent process is needed to support agency and mutual decision‐making. This systematic review aims to gather and characterise the evidence supporting methods that enhance inclusion and accessibility in informed consent practice for people with vision and/or hearing support needs. It will address the research question: what strategies are being used to enhance inclusive consent practice for people with vision impairment, people who are d/Deaf or hard of hearing, and people who are d/Deafblind. Additionally, the review aims to generate recommendations to facilitate the uptake of accessibility practices within informed consent procedures. This systematic review will build on current evidence for the positive impact of intentional actions to support accessible communication and information exchange. It may guide future work on intervention development and primary research in improving equity in clinical care and research.

## 1 Background

People with sensory impairment, encompassing vision and/or hearing difficulties, may have more equitable opportunities to participate in clinical care and research if provided with accessible communication and information. Providing communications and information in an accessible way means that a person is able to locate, read or receive information, and understand it in a way that meets their needs and preferences (RDI Network 2020). Accessibility standards are supported by international and domestic law. For example, *The Equality Act 2010* underpins the *NHS England Accessible Information Standard*, and the *Disability Discrimination Act (1992*) is in place in Australia (Disability Discrimination Act 1992 (Cth)). Across the world, universal law is stipulated in the *United Nations Convention on the Rights of Persons with Disabilities*, which was adopted in 2006 (UN General Assembly 2006). These laws and regulations indicate that human rights values have been in place for over two decades. They recognise that people with disability have the right to dignity and choice in all areas of life, including health, where supports and services need to be accessible and equitable (Griffith 2022). Whilst the term ‘disability’ is an acceptable term used in and out of the disability sector, we have selected to use the term ‘difficulty’ or ‘support needs’ in this paper, specific to the impact on the senses of vision and hearing. This is in keeping with the social and human rights models of disability, where barriers to information and communication, for example, are understood to be imposed by the environment, rather than ascribed to the individual (Retief and Letšosa 2018). Arguably, disability would cease to exist if accessibility standards were universally accepted and implemented.

Implementation of accessibility standards in clinical care and research is important for several reasons. Firstly, supporting people to make adequately informed choices and decisions promotes autonomy, independence, and agency in the process of providing their consent to engage or participate (Hagopian 2024). Second, the more people that participate in clinical research, the better the research outcomes are for all of society, as more diversity is represented. Third, there are disclosed and undisclosed disability in our community, and of note, vision and hearing difficulties increase with age, and the population is ageing (Haile et al. 2021; Li et al. 2022). As such, there is an increasing need to guide meaningful policies and practices that accommodate all capacity levels and abilities.

Despite the positive impacts of adopting accessibility practices, the reality is that people with vision and hearing support needs encounter regular barriers with inaccessible information and communications (Chien and Wu 2024; Owens 2006). In the context of environmental barriers, this may include language that is too complex, text or printed content that is not available in accessible language, and formats such as braille, large print, audio and electronic formats. It also includes not having access to sign and tactile language interpreters. The realisation of these actions requires legislation, capacity building and intentional actions to ensure that universal accessibility is met (Campbell 2008). Within the healthcare and research sectors, evidence suggests there is a general lack of staff awareness, education and training around accessibility strategies suitable for people with vision and hearing support needs (Browne and Dorris 2022; Kuper and Shakespeare 2024; Sabatello et al. 2019). Additionally, what technology and services are available to assist staff in implementing these strategies (Bigby et al. 2023). For informed consent practice, guidelines exist that are produced by the community support organisations, yet there is varied implementation of this expert knowledge in the clinical research sector.

Clinical research teams are responsible for upholding the fundamental principles of informed consent, with the process including the person being provided with information they understand (e.g., good clinical practice guidelines, ICH GCP 2025). For the informed consent process to be inclusive, it should enable accessible information provision and effective communication exchange (Jawa et al. 2023; Kadam 2017). There is a need to identify what accessibility strategies are being employed to engage with people who have vision and hearing difficulties, and in doing so, help raise awareness of their use to support wider uptake in informed consent procedures. Filling this knowledge gap is warranted, and efforts are required to build skills and capacity in offering routine accessibility practices with people who have vision and hearing difficulties (Kushalnagar et al. 2023; Shariq et al. 2023; Tanner et al. 2019).

### 1.1 Why It Is Important to Do This Review

We conducted a preliminary search for systematic and scoping reviews across databases, including the Cochrane Library and Medline, to ensure the research question, concepts and target populations had not been previously investigated. To the best of our knowledge, the only review with a specific focus on informed consent procedures with people with sensory impairments is a scoping review by Paramasivam et al. (2021). More recently, a scoping review by Krawczyk et al. (2024) has focused on research engagement more broadly with people who are d/Deaf and the need for more cultural awareness.

Paramasivam et al. explored informed consent and assent strategies for research involving people with all levels of combined vision and hearing difficulty or Deafblindness. The review also included a second target group, people with intellectual disability. This review focused on a small interval period (January 2015–July 2020) and excluded publications relating to clinical trials. Given the high level of informed consent procedures undertaken in the clinical trial setting, we seek to build on their valuable work and supplement findings to provide greater impact. The themes identified in Paramasivam et al. review covered accessibility, relationship building, behavioural cues and communication training (for researchers). To increase understanding of the needs of people experiencing sensory challenges involving vision and hearing, raise awareness of accessible formats, and expand their use in informed consent practice in clinical trials and clinical care, a broader systematic review is needed.

The planned systematic review will provide clinicians and researchers with valuable education and insights into person‐centred communication practices, to aid them in delivering tailored yet equitable informed consent procedures to support people who experience any level of change to their sensory ability. Whilst the intention is never to categorise people based on a diagnosis of impairment, this review aims to help broaden the understanding that individual or combined changes to vision and hearing will affect a significant proportion of our community. The planned systematic review will ultimately broaden the evidence to help inform policy and inclusive best practice. It will also guide future work on intervention development and primary research in improving equity and inclusion in clinical care and research.

## 2 Objectives

### 2.1 The Main Objectives

This systematic review will collate, describe and characterise the available evidence regarding what accessibility strategies are employed, and describe the outcomes and impacts of these approaches. It will synthesise evidence on the most effective strategies to improve information accessibility and engagement processes with target populations: people with vision impairment encompassing low vision and blindness, hearing difficulty encompassing d/Deaf and hard of hearing, and those with any level of combined vision and hearing loss or d/Deafblindess. These are three distinctive and diverse target populations, and there is recognition that information and communication needs will be unique across and within each population. There is also recognition of a wide range of terms, disability epistemologies, and community preferences for person‐first or identify‐first language. Within this review and for the purpose of referring to all target populations, reference to vision impairment, hearing difficulty and Deafblindness will be made and the collective term ‘sensory impairment’ will be applied to include these target populations. It is recognised that sensory impairments can impact any of the four other senses. However, for the purpose of this review, the definition is ascribed to vision and hearing only.

### 2.2 Research Question

This systematic review will answer the following question:

What strategies are being used to enhance inclusive consent practice for people with sensory impairment? This includes procedural adaptations and approaches to enhance inclusion, accessibility or acceptability during clinical research participation that involves informed consent processes with target populations.

## 3 Methods

The systematic literature review will follow the Preferred Reporting Items for Systematic Reviews and Meta‐Analysis Protocols (PRISMA‐P) (Moher et al. 2015; Page et al. 2021). The review will be conducted following the PRISMA 2020 checklist and ENhancing Transparency in REporting the synthesis of Qualitative research (ENTREQ) reporting guidelines (Tong et al. 2012). Consumer representatives, those with lived experience of vision impairment or hearing difficulty, are involved in this study as research partners and have contributed to this manuscript.

### 3.1 Eligibility Criteria

#### 3.1.1 Study Designs

Studies will be eligible if they use qualitative, quantitative, or mixed methods approaches; examine quantitative or qualitative research outcomes. This could include action research, ethnographic research, descriptive studies, cohort studies, observational research, textual analysis research, surveys, interviews, focus group or workshops.

#### 3.1.2 Types of Participants

Included studies will be required to involve (1) participants with sensory impairment, or (2) participants who are researchers or health providers, or both. For the former, studies involving either children or adults/parents will be included. Studies that specifically focus on the incapacity to provide consent (noting that everyone should be given the opportunity, and very few instances exist where this is not possible) will be excluded.

#### 3.1.3 Types of Strategies or Approaches Taken

Studies are required to examine accessible information and communication strategies for clinical research participation or informed consent processes. They must include primary research data (i.e., opinion pieces and editorials will be excluded).

There are a variety of strategies and approaches that may aid the consent process for people with sensory impairment. Examples may include provision of information in audio‐visual media, verbal consent discussions, use of braille, language, linguistic and tactile interpreters. This is supported by global standards such as the International Council for Harmonisation, which updated the guideline for Good Clinical Practice, recommendation 2.8.1, that states that varied approaches to information provision ‘may’ be adopted, such as using ‘text, images, videos and other interactive methods’ (ICH GCP 2025). Approaches to assisting people with reading and understanding printed information also include producing printed information in a simple layout that is consistent and logical, and presenting text that is legible and well‐spaced (Round Table 2022).

#### 3.1.4 Types of Outcome Measures

Our review will identify the different interventions that have been used and the outcomes reported. A range of outcome measures is likely to be reported with respect to participants/patients and/or clinicians/researchers. Outcome measures from included studies could include factors relating to the implementation of the intervention, or direct implementation outcomes such as acceptability, appropriateness and feasibility. Specific to the informed consent process, outcomes around participant understanding, recall, decision‐making support and participant enrolment are often reported. The review will assess the quality of this evidence and provide a summary of findings.

#### 3.1.5 Types of Settings

Studies that are based in medical, healthcare, clinical research, and clinical trial settings will be included. Studies in any geographical setting (high‐, low‐ and middle‐income countries) will be included.

### 3.2 Information Sources

We will search five electronic databases, Medline (Ovid), Embase (Ovid), PsycInfo (Ovid), CINAHL (EBSCO) and SCOPUS. Google Scholar, a search engine that indexes scholarly literature, will be included as a grey literature source. These sources were selected to cover publications in biomedical science, allied health and medical fields, clinical research, and social sciences.

### 3.3 Search Strategy

A pilot literature search was conducted in August 2024 in Medline (Ovid) and Google Scholar with limited search terms to gather further keyword search terms and subject headings. This process involved two authors who have expertise in databases as librarians.

Multiple search terms for vision impairment, hearing difficulty and Deafblindness were identified and tested on all information resources. Particular focus was on terms for vision impairment, as the term ‘blind’ retrieves double‐blind and single‐blind studies, so other terms were used to compensate.

The second concept consisted of three parts: informed consent (consent); research (design, clinical trial, participation, recruitment, engagement); and inclusion (inclusive research, participatory design, engagement, accessibility).

To cover the concepts of the literature review, a mix of medical, psychology, sociology and allied health databases is selected. The search has been tested and translated for all selected databases, with amendments made for Google Scholar (Clark et al. 2020).

Publications from the year 2000 will set the timeframe for the search and therefore encompass over two decades of insights. The complete search strategy is presented in Supporting Information SA1.

The planned dates for coverage were selected in recognition of advances in digital information technology and assistive technology to enable information access (Gesualdo et al. 2021; Kiernan et al. 2023). Furthermore, this period will capture studies that investigate accessible consent practices, which is a feature of literature in more recent years supported by amendments to best practice guidelines (ICH GCP 2025).

#### 3.3.1 Searching Other Resources

Reference list checking will be performed by manually reviewing and retrieving cited references from seed (included) papers (Hirt et al. 2024). Additionally, the reference lists of key systematic reviews identified in the pilot search phase will be performed. The number of papers from backward and forward citation searching that meet the eligibility criteria will be recorded.

### 3.4 Study Records

#### 3.4.1 Selection and Data Collection Process

The process used for selecting studies will involve a minimum of two independent reviewers throughout each phase of the review. All the results from the electronic database searches and the searches of other resources will be added to Covidence, a software programme for carrying out systematic reviews (available at www.covidence.org). Duplications will be highlighted and removed both by Covidence and manually, through visual inspection, during the screening of results. Screening of the results will be performed in two stages, first by title and abstract, and second by full text. These stages will be performed independently by a minimum of two review authors. At least one author (first author) will screen all studies, with two or more authors combined to produce the second review of all results. Full text review will be performed independently by two authors, with a third author recruited to discuss discrepancies and reach consensus. Full texts will be reviewed with reference to the eligibility criteria, and the main reason for excluding the study will be assigned in Covidence. As required, the authors of an included article will be contacted if information is missing to assist accurate decision‐making, or if the full text cannot be accessed.

#### 3.4.2 Data Extraction and Management

A data extraction and coding template form has been developed in Covidence (see Supporting Information SA2 for the form fields). Data and information will be extracted on study design and setting, sample characteristics, intervention characteristics, outcome measures, results and quality rating of the study (more information below). Two authors will pilot test the template form based on the extraction of three studies and adjust it as necessary in consultation with other review authors. All other review authors who will participate in the data extraction will be trained in the use of the coding template form. Two review authors and a consensus reviewer will be assigned to each eligible study for independent data extraction. Disagreements between two reviewers will be discussed and resolved with consultation with the consensus reviewer, and subject‐matter experts will be consulted when appropriate. These disagreements and their resolution will be reported. Moreover, if an eligible study is published in a language other than English, efforts will be made to translate the article. Finally, where data or important information is missing from a paper, the authors will be directly contacted, and a request will be made to provide the omitted information.

### 3.5 Data Items

No pre‐planned data assumptions and simplifications are expected.

### 3.6 Outcomes and Prioritisation

We anticipate that the included studies will include quantitative, qualitative and mixed methods data. The quantitative outcomes could include cross‐sectional survey data from close‐ended or multiple‐choice questions. This data may be represented by groups that are exposed to either standard informed consent practices (i.e., standard printed information accompanying verbal) or enhanced informed consent practices (i.e., where information is presented in other multimedia formats). Studies may include randomised or quasi‐experimental studies or case‐control studies. Quantitative measurement and statistical analyses will be extracted that support the main findings (Smith and Hasan 2020).

Qualitative methods may include, but are not limited to, rapid ethnography involving semi‐structured interviews, field notes, participant observations, as well as data from narrations, focus groups and interactive workshops. They may include perspectives shared from the patient or participant voice, or from the researcher or clinician voice. Qualitative studies are expected to present traditional qualitative analysis that may include inductive or deductive thematic analysis or alternative methodology (Hamilton and Finley 2019). Themes will be reviewed for specific reference to recommended actions that enable or facilitate accessible information provision.

### 3.7 Assessment of Quality and Risk of Bias in Included Studies

Two systems will be employed to assess the quality of the evidence. The first relies on assessing the quality of the reporting for individual articles using the ‘QualSyst’ tool (Kmet et al. 2004). This ‘within’ article assessment is focused on study design criteria and is explained in more detail below. The second system is to assess the confidence of the recommendations or findings ‘across’ articles using the Grading of Recommendations Assessment, Development and Evaluation (Puhan et al. 2014) system for quantitative research outcomes (Guyatt et al. 2008). This has also been extended to the Confidence in the Evidence from Reviews of Qualitative research (GRADE‐CERQual) approach (Lewin et al. 2015). These GRADE systems are described in the ‘Confidence in cumulative evidence’ section.

The QualSyst method uses a checklist approach with an appraisal of reporting quality based on 14 assessment criteria for quantitative studies (see Table 1), and 10 assessment criteria for qualitative studies (see Table 2). Each criterion will be scored depending on the degree to which the specific criteria was met, with ‘yes’ equalling a score of two, ‘partial’ equating to a score of one, and ‘no’ equating to a score of zero. Not applicable can also be applied to a criterion, and in this instance, no value is given. A total summary score can be calculated by total sum/highest possible score, excluding the number of ‘not applicable’ items. A table with the rating for each criteria, for each included paper, will be shown. A description of applying the quality assessment will be noted in the tool guide provided by the authors (Kmet et al. 2004). Two authors will independently rate each included paper, and an investigation of levels of agreement between raters will be explored.

**Table 1:** QualSyst checklist for assessing the quality of quantitative studies.

#1	Research question or objective sufficiently described?
#2	Study design evident and appropriate?
#3	Method of intervention/comparison group selection or source of information/input variables described and appropriate?
#4	Participant characteristics sufficiently described?
#5	If interventional and random allocation was possible, was it described?
#6	If interventional and blinding of investigators was possible, was it reported?
#7	If interventional and blinding of subjects was possible, was it reported?
#8	Outcome and (if applicable) exposure measure(s) well defined and robust to measurement/misclassification bias? Means of assessment reported?
#9	Sample size appropriate?
#10	Analytic methods described/justified and appropriate?
#11	Some estimate of variance is reported for the main results?
#12	Controlled for confounding?
#13	Results reported in sufficient detail?
#14	Conclusions supported by the results?

*Note:* For the quantitative studies, 14 items (list above) will be scored depending on the degree to which the specific criteria was met (‘yes’ = 2, ‘partial’ = 1, ‘no’ = 0, not applicable = NA). Items rated as NA will be excluded from calculating the summary score.

**Table 2:** QualSyst checklist for assessing the quality of qualitative studies.

#1	Research question or objective sufficiently described?
#2	Study design evident and appropriate?
#3	Context for the study clear?
#4	Connection to a theoretical framework/wider body of knowledge?
#5	Sampling strategy described, relevant and justified?
#6	Data collection methods clearly described and systematic?
#7	Data analysis clearly described and systematic?
#8	Use of verification procedure(s) to establish credibility?
#9	Conclusions supported by the results?
#10	Reflexivity of the account?

*Note:* For the qualitative studies, 10 items (list above) will be scored depending on the degree to which the specific criteria was met (‘yes’ = 2, ‘partial’ = 1, ‘no’ = 0. No items were able to be recorded as not applicable.

In conjunction with the QualSyst method, used to assess the reporting of study design, the confidence and quality of the evidence across studies will be assessed using the GRADE or GRADE‐CERQual method. Consideration will also be given to using the Cochrane risk of bias tool as a pre‐requisite to the GRADE approach for any randomised controlled trials (Jawa et al. 2023).

### 3.8 Confidence in Cumulative Evidence

The Grading of Recommendations Assessment, Development and Evaluation or the GRADE method will be used if the included articles describe quantitative research designs and outcomes (Guyatt et al. 2008). The GRADE method assesses the confidence of each main finding regarding five elements: study limitations, inconsistency of results, indirectness of evidence, imprecision and reporting bias. A four‐tier rating system will be applied: high, moderate, low and very low confidence. The studies contributing to each main finding will be collated in a summary of main findings table.

GRADE is appropriate when looking at intervention effectiveness and is most useful for assessing the quality of evidence from quantitative study designs. As a result, the GRADE CERQual approach has been developed to assess how much confidence can be placed on qualitative research outcomes (Lewin et al. 2015). The GRADE CERQual method will be selected if the included articles are all qualitative study designs. The authors of this approach have produced a series of published papers on how to assess each aspect (Booth et al. 2018; Colvin et al. 2018; Glenton et al. 2018; Lewin, Bohren, et al. 2018; Lewin, Booth, Glenton, et al. 2018a; Munthe‐Kaas et al. 2018; Noyes et al. 2018), as well as a practical guide (Lewin, Booth, Glenton, et al. 2018b). A minimum of two independent raters will apply this method to assess the confidence of each main finding with ratings of high, moderate or low confidence. The method grades four components: methodological limitations, relevance, coherence and adequacy of the study design and outcomes. Irrespective of the system used, the distribution of ratings will be assessed, and review findings that have a low or very low rating on all criteria will be excluded (at the data extraction and analysis stages) based on poor quality of evidence.

### 3.9 Data Synthesis

In addition to the methods described for assessing the quality of the evidence from both quantitative and qualitative syntheses, recommendations may be organised using an implementation science framework. We plan to conduct a qualitative synthesis where findings from qualitative, quantitative and mixed methods studies will be pooled (Bearman and Dawson 2013). Through a series of expert judgements, a unified coding framework will be developed. We anticipate that if inductive coding is most appropriate, we will use a thematic approach. This may help with data familiarisation and coding in an iterative manner. Content analysis will be performed with reference to the aims and objectives of the review. Inductive coding may also be paired with deductive coding. Using a realist review methodology, we will select an implementation science framework as an a priori framework, such as the Consolidated Framework for Implementation Research or the Theoretical Domains Framework (Atkins et al. 2017; Damschroder et al. 2022). An implementation science framework will be helpful in mapping the review's main findings and determining the domain and constructs relevant to the barriers and enablers to implementing strategies to enhance inclusion in informed consent practices. The process of coding both qualitative and quantitative data will be assisted by using Nvivo (provided by the University of Melbourne; https://unimelb.libguides.com/stat_software). Implementing new practices requires changes in the behaviours and actions of the people performing informed consent procedures. Using an implementation framework will help identify strategies and approaches that work at various levels to support changes in policy and practice. Recommendations from this review may then be presented as a final checklist. This will be accompanied by a plain language summary of the findings from the review.

## Author Contributions

Content: Fleur O'Hare, Eden G. Robertson, Sujani Thrimawithana, Aimee Clague, Caroline Ondracek, David Foran, Camille Paynter, Tessa Saunders, Lauren N. Ayton. Systematic review methods: Fleur O'Hare, Sujani Thrimawithana, Aimee Clague, Eden G. Robertson, Caroline Ondracek, Camille Paynter, David Foran. Statistical analysis: Fleur O'Hare, Sujani Thrimawithana, Tessa Saunders, Lauren N. Ayton. Information retrieval: Fleur O'Hare, Sujani Thrimawithana, Aimee Clague, Eden G. Robertson.

## Conflicts of Interest

The authors declare no conflicts of interest.

## Preliminary Timeframe

Approximate time for completion of the review is 1 year from the time the protocol is approved.

## Plans for Updating This Review

Any updates to this protocol will be addressed in the manuscript of the review for publication. Of note, this protocol has been registered on PROSPERO (CRD42024587188, submitted 26 August 2024), and updates will be concurrently updated on this registry in addition to the review manuscript.

## Sources of Support

**Internal sources**

None to report.

**External sources**

F.O.H. is supported by a scholarship from the Australian National Industry PhD Programme (#34984). L.N.A. is supported by a National Health and Medical Research Council Investigator Grant (GNT#1195713). The Centre for Eye Research Australia wishes to acknowledge the support of the Victorian Government through its Operational Infrastructure Support Programme (VIC, Australia).

## Transparent Peer Review

The peer review history for this article is available at https://www.webofscience.com/api/gateway/wos/peer-review/10.1002/cl2.70065.

## Supporting information

Appendix S1.

## Data Availability

The data that supports the planned review of this study will be available in Minerva Access, an open access repository used by the University of Melbourne, Victoria, Australia. Supporting material is included in this manuscript. Supporting Information SA1 is the complete database search. Supporting Information SA2 is the data extraction and coding framework template form.
