# Supplementary material for: Protocol: Strategies to Enhance Inclusion in Informed Consent Practice for People With Vision and/or Hearing Support Needs: A Systematic Review
Source: Campbell Syst Rev. 2025 Sep 29;21(4):e70065. doi: 10.1002/cl2.70065 (PMC12477491; doi:10.1002/cl2.70065)
Supplement: Supplementary file 1 — Appendix S1. [file CL2-21-e70065-s001.docx]

# Appendices

# Appendix 1 - Search Strategy

## A. Database search

The search strategy was developed using subject headings and search terms related to 1) informed consent, 2) research methods to promote engagement and inclusion, and 3) sensory loss, and were combined using the ‘OR’ Boolean operator. Truncated search terms will be used to search titles and key words to capture all possible relevant studies. The search strategy was developed by three authors (FOH, ST and AC). A forth author assisted with database translation (CO). The selected databases to be searched are: OVID Medline, Cumulative Index to Nursing and Allied Health Literature/CINAHL, Scopus, Embase, and PsycInfo. These databases were chosen as they include clinical research and ethical practice across allied health, medical, bioscience, social sciences and health promotion databases. Grey literature will also be searched from Google Scholar.

**B.** Search terms for each database

### **1. OVID Medline**

1. research design/ or patient selection/ or exp clinical trials as topic/
2. research.kf.
3. clinical trial.kf
4. 1, 2 or 3
5. (participation or involvement or recruitment or engagement or accessibl*).tw,kf.
6. 4 and 5
7. ((research or clinical trial) adj5 (participation or involvement or recruitment or engagement or accessibl*)).tw.
8. Inclusive research.mp.
9. Participatory design.mp.
10. consent.mp
11. 6 or 7 or 8 or 10
12. (dual sensory or dual-sensory).mp.
13. (deafblind* OR deaf blind* OR deaf-blind*).mp.
14. (visual* adj (impair* OR disab*)).mp.
15. ((vision or sight*) adj (loss or impair* or disab*)).mp.
16. (partial* or low or reduced adj (vision or sight*)).mp.
17. blindness.mp.
18. (blind adj (person* or people* or communit* or participant* or subject or subjects or user*)).mp.
19. legally blind.mp.
20. individuals who are blind.mp.
21. (hearing adj (loss or impair* or disab*)).mp.
22. ((partial or reduced or impair*) adj hearing).mp
23. (hard adj2 hearing).mp.
24. deaf*.mp.
25. 12 or 13 or 14 or 15 or 16 or 17 or 18 or 19 or 20 or 21 or 22 or 23 or 24
26. 11 and 25
27. Limit range to yr = “2000-Current”

### **2. CINAHL**

1. (MH "research design") OR (MH "patient selection") OR (MH "clinical trials as topic+")

2. (AB research)

3. (AB "clinical trial")

4. S1 OR S2 OR S3

5. ((TI participation OR AB participation OR SU participation) OR (TI involvement OR AB involvement OR SU involvement) OR (TI recruitment OR AB recruitment OR SU recruitment) OR (TI engagement OR AB engagement OR SU engagement) OR (TI accessibl* OR AB accessibl* OR SU accessibl*))

6. S4 AND S5

7. (((TI research OR AB research) OR (TI "clinical trial" OR AB "clinical trial")) N4 ((TI participation OR AB participation) OR (TI involvement OR AB involvement) OR (TI recruitment OR AB recruitment) OR (TI engagement OR AB engagement) OR (TI accessibl* OR AB accessibl*)))

8. "inclusive research"

9. "participatory design"

10. consent

11. S6 OR S7 OR S8 OR S9 OR S10

12 . ("dual sensory" OR dual-sensory)

13. (deafblind* OR "deaf blind*" OR deaf-blind*)

14. (visual* W0 (impair* OR disab*))

15. ((vision OR sight*) W1 (loss OR impair* OR disab*))

16. (partial* OR low OR reduced) W1 (vision OR sight*)

17. blindness

18. (blind W1 (person* OR people* OR communit* OR participant* OR subject OR subjects OR user*))

19. "legally blind"

20. "individuals who are blind"

21. (hearing W1 (loss OR impair* OR disab*))

22. ((partial OR reduced OR impair*) W1 hearing)

23. (hard N1 hearing)

24. deaf*

25 . S12 OR S13 OR S14 OR S15 OR S16 OR S17 OR S18 OR S19 OR S20 OR S21 OR S22 OR S23 OR S24

26 . S11 AND S25

27. Limiters - Publication Date: 20000101-20241231

Search modes - Proximity

### **3. Scopus**

1. INDEXTERMS ( “research design” ) OR INDEXTERMS ( “patient selection” ) OR INDEXTERMS ( “clinical trials as topic” )

2. AUTHKEY ( research ) OR AUTHKEY ( "clinical trial" )

3. 1 OR 2

4. TITLE-ABS-KEY ( participation OR involvement OR recruitment OR engagement OR accessibl* )

5. 4 AND 3

6. TITLE-ABS-KEY ( "inclusive research" OR "participatory design" OR consent )

7. TITLE-ABS ( ( research OR “clinical trial” ) W/4 ( participation OR involvement OR recruitment OR engagement OR accessibl* ) )

8. 5 OR 6 OR 7

9. TITLE-ABS-KEY ( hard W/1 hearing )

10. TITLE-ABS-KEY ( partial OR reduced OR impair* ) PRE/0 hearing

11. TITLE-ABS-KEY ( hearing PRE/0 ( loss OR impair* OR disab* ) )

12. TITLE-ABS-KEY ( "individuals who are blind" )

13. TITLE-ABS-KEY ( "legally blind" )

14. TITLE-ABS KEY ( blind PRE/0 ( person* OR people* OR communit* OR participant* OR subject OR subjects OR user* ) )

15. TITLE-ABS-KEY ( blindness )

16. TITLE-ABS-KEY ( ( partial* OR low OR reduced ) PRE/0 ( vision OR sight* ) )

17. TITLE-ABS-KEY ( ( vision OR sight* ) PRE/0 ( loss OR impair* OR disab* ) )

18. TITLE-ABS-KEY ( visual* PRE/0 ( impair* OR disab* ) )

19. TITLE-ABS-KEY ( deafblind* OR deaf-blind* OR "deaf blind*" )

20. TITLE-ABS-KEY ( "dual sensory" OR dual-sensory )

21. INDEXTERMS ( "Visually Impaired Persons" OR deafness OR "Persons With Hearing Impairments" OR "deaf-blind disorders" )

22. 8 AND 21

23. PUBYEAR > 1999

**4. Embase**

1. methodology/ or patient selection/ or exp "clinical trial (topic)"/

2. research.kf.

3. clinical trial.kf.

4. 1 or 2 or 3

5. (participation or involvement or recruitment or engagement or accessibl*).tw,kf.

6. 4 and 5

7. ((research or clinical trial) adj5 (participation or involvement or recruitment or engagement or accessibl*)).tw.

8. inclusive research.mp.

9. participatory design.mp.

10. consent.mp.

11. 6 or 7 or 8 or 9 or 10

12. (dual sensory or dual-sensory).mp.

13. (deafblind* or deaf-blind* or deaf blind*).mp.

14. (visual* adj (impair* or disab*)).mp.

15. ((vision or sight*) adj (loss or impair* or disab*)).mp.

16. ((partial* or low or reduced) adj (vision or sight*)).mp.

17. blindness.mp.

18. (blind adj (person* or people* or communit* or participant* or subject or subjects or user*)).mp.

19. legally blind.mp.

20. individuals who are blind.mp.

21. (hearing adj (loss or impair* or disab*)).mp.

22. ((partial or reduced or impair*) adj hearing).mp.

23. (hard adj2 hearing).mp.

24. deaf*.mp.

25. 12 or 13 or 14 or 15 or 16 or 17 or 18 or 19 or 20 or 21 or 22 or 23 or 24

26. 11 and 25

27. limit 26 to yr="2000 -Current"

**5. PsychInfo**

1. experimental design/ or patient selection/ or clinical trials/

2. research.id.

3. clinical trial.id.

4. 1 or 2 or 3

5. (participation or involvement or recruitment or engagement or accessibl*).tw,id.

6. 4 and 5

7. ((research or clinical trial) adj5 (participation or involvement or recruitment or engagement or accessibl*)).tw.

8. inclusive research.mp.

9. participatory design.mp.

10. consent.mp.

11. 6 or 7 or 8 or 9 or 10

12. (dual sensory or dual-sensory).mp.

13. (deafblind* or deaf-blind* or deaf blind*).mp.

14. (visual* adj (impair* or disab*)).mp.

15. ((vision or sight*) adj (loss or impair* or disab*)).mp.

16. ((partial* or low or reduced) adj (vision or sight*)).mp.

17. blindness.mp.

18. (blind adj (person* or people* or communit* or participant* or subject or subjects or user*)).mp.

19. legally blind.mp.

20. individuals who are blind.mp.

21. (hearing adj (loss or impair* or disab*)).mp.

22. ((partial or reduced or impair*) adj hearing).mp.

23. (hard adj2 hearing).mp.

24. deaf*.mp.

25. 12 or 13 or 14 or 15 or 16 or 17 or 18 or 19 or 20 or 21 or 22 or 23 or 24

26. 11 and 25

27. limit 26 to yr="2000 -Current"

**6. Google Scholar**

inclusive research|"participatory design"|consent|"accessible AROUND(5) research" AND "dual sensory"|deafblind*|"deaf blind"|"visual* AROUND(5) impair*"|blindness|"blind person"|"blind community|"hearing loss"|hearing impairment|deaf|deafness

# Appendix 2: Supplement 2 – Data Extraction and Coding Framework.

The following data will be extracted from the studies that will be included for the synthesis.

## Bibliographic data:

- First Author (Surname, initial).
- Paper title.
- Full citation including journal name, issue, volume.
- Authors with lived experience of sensory impairment or disability (reported/not reported).
- Year of publication or report.
- Country.
- Publication type: journal, book, book chapter, dissertation thesis, conference paper, other.
- DOI if available or URL.

## Study design characteristics:

- Type of study design: qualitative, quantitative, mixed.
- Setting: clinical research, clinical trial, medical/healthcare, community, research institute.
- Description of the informed consent procedure undertaken with the participants in the study. Confirm what accessible practices, if any, were implemented and evaluated.

### Quantitative data:

- Sampling: simple random, systematic, stratified, clustered, quota, snowballing, convenience, probability.
- Type of design: random, quasi-experimental, non-random.
- Number of control or comparator groups.

### Qualitative data:

- Sampling: convenience, snowballing, purposive, key informant.
- Type of design: interviews, narrations or personal stories, focus groups, meetings or community consultations, workshops, vignettes, storyboards, other (specify).
- Time points: single, multiple (specify).

### Participant data:

- Number of participants, within and across groups as relevant.
- Age, gender and ethnicity or culture.
- Disability or impairment category (vision, hearing or vision and hearing).

## Study Outcomes

- Primary aim of the study.
- Data collection methods.
- What is being measured? Intervention type/s. Refer to protocol section 3.1.3.
- What are the key outcomes? Primary outcome variable or main theme/s identified (top tier coding). Refer to protocol section 3.1.3.
- Secondary outcome variable/s or subthemes identified.
- What are the main outputs? For example, checklist, guide, recommendations.

## Results

- For each criteria, score of yes, no, partial or not applicable. This data will be shown in table form for each model.
- Total summary score also calculated for internal analysis only but will not be reported in lieu of showing the rating for each criteria in table form.

See more detailed information below.

### Review results - quality of studies included:

The quality of the reporting of study design for each included study or paper will be assessed using the QualSyst tool (Kmet et al., 2004). The method uses a checklist approach with an appraisal of reporting quality based on 14 assessment criteria for quantitative studies (see Table 1 presented in text), and 10 assessment criteria for qualitative studies (see Table 2 presented in text).

Each criterion will be scored depending on the degree to which the specific criteria was met with “yes” equalling a score of two, “partial” equating to a score of one, and “no” equating to a score of zero. Not applicable can also be applied to a criterion and in this instance, no value given. Results will be reported in a table that shows the rating for each criteria per paper. A separate table will be shown for qualitative and quantitative research designs.
